# Current status of intensive end-of-life care in children with hematologic malignancy: a population-based study

**DOI:** 10.1186/s12904-021-00776-5

**Published:** 2021-06-07

**Authors:** Nobuyuki Yotani, Daisuke Shinjo, Motohiro Kato, Kimikazu Matsumoto, Kiyohide Fushimi, Yoshiyuki Kizawa

**Affiliations:** 1 Department of Palliative Medicine, National Centre for Child Health and Development, 2-10-1, Okura, Setagaya-ku, Tokyo, Japan; 2 Department of Information Technology and Management, National Centre for Child Health and Development, Tokyo, Japan; 3 Children’s Cancer Center, National Centre for Child Health and Development, Tokyo, Japan; 4 Department of Health Policy and Informatics, Tokyo Medical and Dental University Graduate School, Tokyo, Japan; 5 Kobe University Graduate School of Medicine, Kobe, Hyogo Japan

**Keywords:** Quality of life, ICU admission, Cardiopulmonary resuscitation, Extra-corporeal membrane oxygenation, Intravenous chemotherapy, Mechanical ventilation, Blood transfusion

## Abstract

**Background:**

Adult patients with hematologic malignancies are less likely to receive palliative care and more likely to accept intensive anti-cancer treatments until end-of-life than those with solid tumors, but limited data are available regarding the quality of end-of-life care (EOLC) for children with hematologic malignancies. To improve the quality of EOLC for children with hematologic malignancies, the aims of this study were (i) to compare intensive EOLC between children with hematologic malignancies and those with solid tumors; and (ii) to describe factors associated with intensive EOLC in children with hematologic malignancies.

**Methods:**

We retrospectively reviewed 0- to 18-year-old patients with cancer, who died in hospital between April 2012 and March 2016 in Japan using the Diagnosis Procedure Combination per-diem payment system. Indicators of intensive inpatient EOLC were defined as intensive care unit admission, cardiopulmonary resuscitation (CPR), intubation and/or mechanical ventilation, hemodialysis, or extra-corporeal membrane oxygenation in the last 30 days of life, or intravenous chemotherapy in the last 14 days. We determined factors associated with intensive EOLC using regression models. Data regarding use of blood transfusion were also obtained from the database.

**Results:**

Among 1199 patients, 433 (36%) had hematological malignancies. Children with hematologic malignancies were significantly more likely than those with solid tumors to have intubation and/or mechanical ventilation (37.9% vs. 23.5%), intensive care unit admission (21.9% vs. 7.2%), CPR (14.5% vs. 7.7%), hemodialysis (13.2% vs. 3.1%) or extra-corporeal membrane oxygenation (2.5% vs. 0.4%) in their last 30 days, or intravenous chemotherapy (47.8% vs. 18.4%; all *P* < .01) within their last 14 days of life. Over 90% of children with hematological malignancies received a blood transfusion within the last 7 days of life. For hematological malignancies, age under 5 years was associated with CPR and ≥ 2 intensive EOLC indicators. Longer hospital stays had decreased odds of ≥ 2 intensive EOLC indicators.

**Conclusion:**

Children with hematologic malignancies are more likely to receive intensive EOLC compared to those with solid tumors. A younger age and shorter hospital stay might be associated with intensive EOLC in children with hematologic malignancies.

## Background

Hematologic malignancies are the most frequent type of cancer in children, accounting for over 40% of childhood cases. The outcomes for children with hematologic malignancies have dramatically improved over the last several decades and the 5-year survival rate of hematologic malignancy is about 80%-90% in high-income countries [1, 2]. However, about 10%-20% of children will die due to these malignancies.

Many adults who are diagnosed with malignancies may express their wish to avoid life-extending measures at their end-of-life (EOL) [3, 4]. However, in the adult setting, patients with hematologic malignancies are less likely to receive palliative care [5] and more likely to accept intensive anti-cancer treatments until EOL, which is different than for patients with solid tumors [6]. Transfusions and other therapies may result in difficulty in admitting to a hospice [7, 8], creating a burden for those staying at home [9], and may result in admission to hospital until death. Compared to death at home, hospital deaths pose a risk of providing a suboptimal quality of life for patients and an increased potential for a less-than-optimum grief process for caregivers [10–12]. Thus, hematologic malignancies may have more factors that influence EOL decision-making than other cancers.

Children with advanced cancer are more likely to receive intensive treatment at their EOL than adults [13], and indeed, there are some studies reporting that hematologic malignancies are more likely to receive intensive treatment [14–16]. To improve the quality of EOL care (EOLC) for children with hematologic malignancies, it is important to clarify the actual status of EOLC and what factors lead to intensive treatment in order to consider future EOL planning.

The aims of this national Japanese study were (i) to compare intensive EOLC between children with hematologic malignancies and those with solid tumors in Japan; and (ii) to describe factors associated with intensive EOLC in children with hematologic malignancies. The findings will lead to the creation of guidelines for future EOL planning in children with hematological malignancies.

## Methods

### Data source

In this retrospective cohort study, data were extracted from the Diagnosis Procedure Combination per-diem payment system (DPC/PDPS), a national inpatient database in Japan. The DPC/PDPS is a case-mix patient classification system that is linked to payments at acute-care and mixed-care hospitals in Japan. By 2016, more than 1,600 hospitals have adopted the DPC/PDPS-based reimbursement system, accounting for more than half of the 894 000 beds in the country [17].

Anonymous clinical and administrative claims data were collected annually for patients from the participating hospitals. Clinical data were collected based on baseline patient characteristics, diagnosis (based on International Statistical Classification of Diseases and Related Health Problems, 10th revision [ICD-10]), major or minor procedures, medications, use of medical materials, admission and discharge information such as the reason for admission, discharge disposition and outcome at the hospital discharge. The DPC/PDPS also includes information about the hospital, such as the number of beds.

The Institutional Review Board of the Tokyo Medical and Dental University and the National Center for Child Health and Development approved this study. The boards determined that the requirement for informed consent from patients was unnecessary because the data were anonymous.

### Participants and variables

The study population consisted of patients in the DPC database with an oncologic diagnosis, aged 0 to 18 years at the time of their death, who died between April 2012 and March 2016 (fiscal year 2012 to 2015), for whom accurate data were available. These data are only applicable to children who died in hospital. An oncologic diagnosis was defined by ICD-10, including malignant neoplasms (C00-C97), but excluding in situ neoplasms (D00-D09) and benign neoplasms (D10-D36). We could not use the International Classification of Diseases for Oncology (ICD-O-3) due to the unavailability of data.

We collected the data, including age, sex, admission status (planned or unplanned), and cancer type (excluding cured diseases), according to ICD-10 from the database. Age at death was categorized into 4 groups according to a previous study [13]: 0–4, 5–9, 10–14 and 15–18 years. Cancer type was categorized based on hematologic malignancies (C80-C97), CNS tumors (C70-C72), and other solid tumors (other ICD-10 codes). To compare hematologic malignancies with other cancers, CNS tumors and other solid tumors were considered together in the analysis. Hospital characteristics included length of hospital stay (0–29, 30–119, 120 + days), hospital type (university hospital or other) and number of childhood deaths per year (1–5, 6–11, 12–16, 17 +). Data regarding use of intensive care unit (ICU), cardiopulmonary resuscitation (CPR), intubation, mechanical ventilation, hemodialysis, extra-corporeal membrane oxygenation (ECMO), chemotherapy, and blood transfusion were also obtained from the database.

We defined indicators of intensive inpatient EOLC according to previous studies in adults and pediatric patients with cancer [13, 18–21] and after discussions with two oncologists, two palliative care physicians, and one statistician. The indicators were ICU admission, CPR, intubation and/or mechanical ventilation, hemodialysis, or ECMO in the last 30 days of life, or intravenous chemotherapy in the last 14 days of life.

### Statistical analysis

Continuous variables are expressed as mean ± standard deviation (SD) or median and interquartile range (IQR), depending on the overall variable distribution. Group comparisons between hematological malignancies and solid tumors were made using the Pearson’s Chi-squared test and Kruskal–Wallis test. Differences in intensive treatment between hematologic and solid tumors were compared using univariate analysis. For children with hematological malignancies, multilevel logistic regression models were constructed to determine factors associated with: (1) ≥ 1 intensive EOLC indicator; (2) ≥ 2 intensive EOLC indicators; (3) CPR; and (4) intravenous chemotherapy. Statistical analyses were conducted using R statistical software v 3.3.2 (R Foundation for Statistical Computing, Vienna, Austria).

## Results

### Participant characteristics

Table 1 shows the characteristics of the study population. Among 1199 patients, 433 (36%) had hematological malignancies. The mean age (SD) of children with hematological malignancies and solid tumors was 10.3 (6.2) and 10.3 (5.8) years, respectively. We found no significant difference in hospital characteristics between children with hematological malignancies and those with solid tumors except for the length of hospital stay (Table 1).

**Table 1:** Characteristics of study patients and hospitals

	All^a^ (*n* = 1199)	Hematologic malignancy^a^ (*n* = 433)	Solid tumor^a^ (*n* = 766)	*P*-value
**Patient characteristics**				
Age at death (years)				.07
0–4	253 (21.1)	100 (23.1)	153 (20.0)	
5–9	266 (22.2)	78 (18.0)	188 (24.5)	
10–14	263 (21.9)	97 (22.4)	166 (21.7)	
15–18	417 (34.8)	158 (36.5)	259 (33.8)	
Sex, female	529 (44.1)	175 (40.4)	354 (46.2)	.06
Cancer type				
Hematologic malignancy	433 (36.1)	433 (100)	0 (0)	
CNS tumor	307 (25.6)	0 (0)	307 (40.1)	
Other solid tumor	459 (38.3)	0 (0)	459 (59.9)	
Urgent admission	245 (20.4)	65 (15.0)	180 (23.5)	< .01
**Hospital characteristics**				
Hospital type				.40
University hospital	765 (63.8)	269 (62.1)	496 (64.8)	
Other hospital	434 (36.2)	164 (37.9)	270 (35.2)	
Length of hospital stay (days)				< .01
0–29	401 (33.4)	94 (21.7)	307 (40.1)	
30–119	408 (34.0)	147 (33.9)	261 (34.1)	
120 +	390 (32.5)	192 (44.3)	198 (25.8)	
Number of childhood deaths per year				.34
1–5	298 (24.9)	101 (23.3)	197 (25.7)	
6–11	301 (25.1)	110 (25.4)	191 (24.9)	
12–16	306 (25.5)	104 (24.0)	202 (26.4)	
17 +	294 (24.5)	118 (27.3)	176 (23.0)	
Year of death				.53
2012–2013	607 (50.6)	225 (52.0)	382 (49.9)	
2014–2015	592 (49.4)	208 (48.0)	384 (50.1)	

^a^Data indicate number of patients (%), unless otherwise indicated

### Intensive EOLC indicators for children with hematologic malignancies compared to those with solid tumors

The most common intensive EOLC indicators were intravenous chemotherapy (29.0%) and intubation and/or mechanical ventilation (28.7%). We found significant differences in the intensive EOLC indicators between children with hematologic malignancies and those with solid tumors (Table 2). In the last 30 days of life, children with hematologic malignancies were significantly more likely to have intubation and/or mechanical ventilation (37.9% vs. 23.5%; *P* < 0.01), ICU admission (21.9% vs. 7.2%; *P* < 0.01), CPR (14.5% vs. 7.7%; *P* < 0.01), hemodialysis (13.2% vs. 3.1%; *P* < 0.01), or ECMO (2.5% vs. 0.4%; *P* < 0.01), than those with solid tumors. In addition, children with hematological malignancies were significantly more likely to receive intravenous chemotherapy within the last 14 days of life (47.8% vs. 18.4%; *P* < 0.01). The composite score for intensive EOLC indicators was significantly higher for children with hematological malignancies compared to those with solid tumors (*P* < 0.01).

**Table 2:** High-intensity end-of-life care for children with hematologic malignancies compared to solid tumors

	All^a^ (*n* = 1199)	Hematologic malignancy^a^ (*n* = 433)	Solid tumor^a^ (*n* = 766)	*P*-value
**Last 30 days of life**				
Intubation and/or mechanical ventilation	344 (28.7)	164 (37.9)	180 (23.5)	< .01
ICU admission	150 (12.5)	95 (21.9)	55 (7.2)	< .01
Cardiopulmonary resuscitation	122 (10.1)	63 (14.5)	59 (7.7)	< .01
Hemodialysis	81 (6.8)	57 (13.2)	24 (3.1)	< .01
ECMO	14 (1.2)	11 (2.5)	3 (0.4)	< .01
**Last 14 days of life**				
Intravenous chemotherapy	348 (29.0)	207 (47.8)	141 (18.4)	< .01
Total No. intensity indicators				< .01
0	571 (47.6)	122 (28.2)	449 (58.6)	
1	367 (30.6)	150 (34.6)	217 (28.3)	
2	151 (12.6)	80 (18.5)	71 (9.3)	
3	69 (5.8)	52 (12.0)	17 (2.2)	
4	36 (3.0)	25 (5.8)	11 (1.4)	
5	5 (0.4)	4 (0.9)	1 (0.1)	

*Abbreviations*: *ICU* Intensive care unit, *ECMO* Extra-corporeal membrane oxygenation

^a^Data indicate number of patients (%), unless otherwise indicated

### Blood transfusions at EOL

Children with hematological malignancies were significantly more likely to receive a blood transfusion within the last 14 days of life, the last 7 days of life, or even within the last 24 h of life, than those with solid tumors. Over 90% of children with hematological malignancies received a blood transfusion within the last 7 days of life, and about 35% of these children received one within the last 24 h of life (Table 3).

**Table 3:** Blood transfusions at end-of-life

**Blood Transfusion**	All^a^ (*n* = 1199)	Hematologic malignancy^a^ (*n* = 433)	Solid tumor^a^ (*n* = 766)	*P*-value
Last 14 days	738 (61.7)	405 (93.5)	333 (43.5)	< .01
Last 7 days	688 (57.4)	398 (91.9)	290 (37.9)	< .01
Last 24 h	224 (18.7)	151 (34.9)	73 (9.5)	< .01

^a^Data indicate number of patients (%), unless otherwise indicated

### Factors associated with intensive EOLC in children with hematologic malignancies

In a multivariate regression analysis, ≥ 1 intensive EOLC indicator, ≥ 2 intensive EOLC indicators, and CPR were associated with young age (0–4 years) in children with hematological malignancies (Table 4). For patients 0–4 years old (vs. patients 15–19 years old) the odds of 2 or more intensive EOLC indicators was 2.3-fold higher. In particular, the odds of CPR was higher in the younger age group. Longer hospital stays were associated with a lower odds of 2 or more intensive EOLC indicators.

**Table 4:** Adjusted odds ratios for receiving high-intensity end-of-life care in children with hematologic malignancies

Category (Reference)	≥ 1 intensity indicator		≥ 2 intensity indicators		Cardiopulmonary resuscitation		Intravenous chemotherapy	
	aOR	*P*-value	aOR	*P*-value	aOR	*P*-value	aOR	*P*-value
Age, years (15–19)								
0–4	**2.11 (1.09–4.08)**	**.03**	**2.33 (1.30–4.20)**	** < .01**	**5.41 (2.20–13.28)**	** < .01**	1.06 (0.61–1.83)	.84
5–9	1.01 (0.53–1.91)	.98	1.19 (0.63–2.23)	.59	**4.21 (1.67–10.61)**	** < .01**	0.94 (0.52–1.70)	.84
10–14	1.22 (0.66–2.24)	.53	1.42 (0.79–2.56)	.24	**3.26 (1.27–8.37)**	**.01**	1.17 (0.67–2.03)	.58
Sex (Male)								
Female	0.89 (0.56–1.41)	.89	0.80 (0.51–1.23)	.31	0.74 (0.40–1.37)	.34	0.84 (0.56–1.27)	.40
Admission (Non-Urgent)								
Urgent	1.24 (0.64–2.41)	.52	1.06 (0.57–1.96)	.85	0.39 (0.13–1.21)	.10	0.75 (0.42–1.36)	.34
Length of hospital stay, days (0–29)								
30–119	1.12 (0.61–2.08)	.71	0.61 (0.34–1.09)	.10	0.59 (0.26–1.35)	.21	0.76 (0.44–1.33)	.34
120-	1.19 (0.66–2.14)	.57	**0.50 (0.28–0.88)**	**.02**	0.76 (0.35–1.63)	.48	0.65 (0.38–1.11)	.11
Hospital type (Other hospital)								
University hospital	1.47 (0.84–2.58)	.19	1.44 (0.84–2.46)	.19	1.06 (0.52–2.17)	.87	1.40 (0.85–2.32)	.19
Number of deaths per year in hospital (1–5)								
6–11	1.71 (0.84–3.48)	.14	1.05 (0.54–2.07)	.88	1.15 (0.46–2.89)	.76	1.68 (0.88–3.18)	.12
12–16	1.68 (0.78–3.63)	.19	0.87 (0.42–1.79)	.70	1.05 (0.39–2.82)	.93	1.73 (0.87–3.44)	.12
17-	1.24 (0.59–2.61)	.58	0.70 (0.33–1.45)	.33	0.57 (0.20–1.62)	.29	1.21 (0.61–2.40)	.58
Year (2012–2013)								
2014–2016	1.18 (0.75–1.86)	.48	1.20 (0.78–1.85)	.40	1.15 (0.64–2.08)	.65	1.23 (0.82–1.85)	.32

*Abbreviation*: *aOR* Adjusted odds ratio

## Discussion

To our knowledge, this study is the first national survey to examine the frequency of intensive EOLC for children with hematologic malignancies, compared to solid tumors.

We found that children with hematologic malignancies are more likely to receive intensive EOLC than those with solid tumors in Japan. Our results reveal that just over half of children (52.4%) with cancer receive at least one measure of intensive EOLC, whereas over two-thirds of children (71.8%) with hematologic malignancies receive at least one measure of intensive EOLC. Hematologic malignancies have a higher death rate from treatment-related complications than solid tumors [22], so patients with hematological malignancies have a higher rate of ICU admissions due to treatment-related complications, such as acute respiratory failure and shock, compared to those with solid tumors [23, 24]. In our study, about one in five children with hematological malignancies used the ICU within 30 days of death. Furthermore, hematologic malignancies respond better to chemotherapy, so hematologic oncologists are more likely than solid-tumor oncologists to recommend chemotherapy [25, 26]. Compared to a population-based study of adults with hematologic malignancies [6], our pediatric patients received more intravenous chemotherapy within 14 days of death than the adult patients (47.8% vs. 21%, respectively), which might reflect the higher sensitivity of pediatric hematologic malignancies even at an advanced stage. Hematologic malignancies, even in the relapsed setting, are often highly treatable when compared to relapsed solid tumors. Good treatment responsiveness makes prognosis difficult to estimate. In Japan, it is difficult to provide chemotherapy at home, and it is also difficult to be admitted to a hospice if you are undergoing chemotherapy, which may have resulted in children having to choose hospital treatment until the end. On the other hand, the fact that the hospital stay was longer for pediatric patients with hematological malignancies than for those with solid cancers may also be due to the effects of chemotherapy. Patients with solid tumors who die in hospitals may be more likely to die after a series of short hospitalizations for treatment. In pediatric hematologic malignancies, palliative care specialists and oncologists need to work together to understand the unique needs of children and develop models of concurrent palliative care [27].

We also found that over 90% of children with hematologic malignancies received blood transfusions within 14 days of death. When compared with adults with hematologic malignancies [28], our results revealed a much higher rate of blood transfusions (adult, 41.7%; pediatric, 93.5%) within 14 days of death, which might reflect stronger myelosuppression. At EOL, blood transfusions may be considered a way to improve quality of life [29, 30]. However, providing a blood transfusion may prevent the option of dying at home or in a hospice [31] or contribute to prolonging life against the patients’ own will. In Japan, there are only a limited number of hospices and home settings where blood transfusions are available. It is important to consider whether a transfusion is really needed and truly leads to symptom relief, and to avoid using blood transfusions just as a measure to sustain life. It is also important to establish a system where transfusions for alleviation of symptoms can be performed outside hospitals.

The third important finding of our study identifies factors associated with intensive EOLC in children with hematologic malignancies. Children under 5 years of age are more likely to receive intensive EOLC, and CPR specifically; this is similar to reports from California [20]. While parents who choose hospice care report being influenced by their child's wishes [32], intensive EOLC in young children may be the result of their inability to express their wishes. Being in hospital over 120 days has a lower odds of 2 or more intensive indicators, suggesting that for those with long anticipated hospital stays, EOL discussions may limit unnecessary intensive care.

The present study has several limitations. First, because we used the national inpatient database, children who died outside the hospital setting were not included in the target population for this study. Children who die outside hospital are likely to receive less intensive treatment and blood transfusion, and in fact may receive a lower percentage of intensive treatment and blood transfusion. Second, because we did not have the opinions of patients and families regarding EOLC, we do not know what types of EOLC patients and families want. Future research on the EOLC desired by children and their families is greatly needed. Third, because we could not determine which children received palliative care services, we could not assess whether receiving palliative care affected the intensive EOLC. And fourth, this was a retrospective study design that precluded interpretations of causality, so we could not tell if hematologic malignancies directly caused receipt of more intensive EOLC.

Based on these results, it is necessary to establish a system in which the palliative care team can intervene from an earlier stage to share the disease status and support decision-making in pediatric hematologic malignancies. At the same time, it is also important to establish a place where treatment, including blood transfusions, can be provided to alleviate symptoms specific to hematologic malignancies.

## Conclusion

Children with hematologic malignancies are more likely to receive intensive EOLC compared to those with solid tumors. A younger age and shorter hospital stay may be associated with intensive EOLC in children with hematologic malignancies. The next step is to clarify how intensive EOLC affects the children's quality of life and the grief of their bereaved families through conducting national surveys of bereaved relatives.

## Data Availability

The datasets used and/or analyzed during the current study are available from the corresponding author on reasonable request.

## Abbreviations

EOL: End-of-life
EOLC: End-of-life care
DPC/PDPS: Diagnosis Procedure Combination per-diem payment system
ICU: Intensive care unit
CPR: Cardiopulmonary resuscitation
ECMO: Extra-corporeal membrane oxygenation
